# The rite of passage of becoming a humanitarian health worker: experiences of retention in Sweden

**DOI:** 10.1080/16549716.2017.1417522

**Published:** 2018-01-15

**Authors:** Sara Albuquerque, Anneli Eriksson, Helle M. Alvesson

**Affiliations:** ^a^ Department of Public Health Sciences, Global Health, Karolinska Institutet, Stockholm, Sweden

**Keywords:** Humanitarian health worker, retention, motivation, rites of passage, self-determination theory, Médecins Sans Frontières

## Abstract

**Background**: Low retention of humanitarian workers poses constraints on humanitarian organisations’ capacity to respond effectively to disasters. Research has focused on reasons for humanitarian workers leaving the sector, but little is known about the factors that can elucidate long-term commitment.

**Objective**: To understand what motivates and supports experienced humanitarian health workers to remain in the sector.

**Methods**: Semi-structured interviews were conducted with 10 experienced nurses who had been on at least three field missions with Médecins Sans Frontières Sweden. Interviews explored factors influencing the decision to go on missions, how nurses were supported and how they looked back on those experiences. Transcripts were analysed through content analysis informed by van Gennep’s concept of ‘Rite of Passage’, combined with elements of the self-determination theory.

**Results**: The findings indicate that their motivations and how nurses thought of themselves, as individuals and professionals, changed over time. For initiation and continued engagement in humanitarian work, participants were motivated by several personal and professional ambitions, as well as altruistic principles of helping others. When starting their first humanitarian missions, nurses felt vulnerable and had low self-esteem. However, through experiencing feelings of autonomy, competence and relatedness during missions, they underwent a process of change and gradually adjusted to new roles as humanitarian health workers. Reintegration in their home community, while maintaining the new roles and skills from the missions, proved very challenging. They individually found their own ways of overcoming the lack of social support they experienced after missions in order to sustain their continuation in the sector.

**Conclusions**: The findings highlight the importance of social environments that facilitate and support the adjustment of individuals during and after field missions. Learning from positive examples, such as nurses with several years of experience, can strengthen strategies of retention, which can ultimately improve the delivery of humanitarian assistance.

## Background

Qualified staff is important for the capacity of humanitarian organisations to respond to disasters. One of the major challenges for humanitarian organisations is a high employee turnover rate [1–4]. Staff turnover leads to loss of experience, skills and organisational memory. It bears direct financial costs, including those from recruitment, training and travels of the new worker, but also costs associated with lower efficiency [1]. In previous studies on staff retention the focus has been to identify reasons for initiating humanitarian work [5,6] and to illustrate the various constraints that humanitarian workers face [1,6–8]. Findings suggest that initiation in humanitarian missions is associated with a wish to assist others in need, a search for new experiences and the opportunity to test personal limits [5,6]. Reasons of personal growth and the need to attain feelings of worthiness have also been identified [5]. Reported hardships among experienced humanitarian workers include those associated with the demanding working and living conditions during missions, such as issues with security, isolation or cultural barriers [6–9]. Those working in the field are often challenged by the close interlink between their professional and private lives [9]. Family obligations, the limited financial support and the restricted career prospects are also sources of concern [1,8]. Two quantitative studies assessed factors which significantly influence turnover in the sector [2,3]. In Médecins Sans Frontières (MSF) Holland, Korff et al. [2] identified higher turnover rates among medical doctors, workers in a permanent relationship or with attractive career opportunities in their home countries. In a later study, Dubey et al. [3] expanded on the number of variables investigated to explore turnover factors in the context of a humanitarian organisation in India. Employee’s perception of the job and a number of individual characteristics, such as marital status and education, were reported. Work-related factors, including role clarification and pay, were shown to be particularly significant in employee turnover [3].

A significant body of literature is dedicated to the motivations and determinants of volunteerism across a range of activities [10–13]. Yet, existing studies tend to concentrate on those who serve in their own countries and without monetary reward [10–12]. In previous literature on aid work, the author critically notes the need to investigate personal accounts and experiences of international aid workers more thoroughly, highlighting its relevance to the delivery of good aid [9]. The understanding of why some humanitarian workers, in spite of adversities, remain in the sector and continue to serve internationally in areas afflicted by disaster, is limited. Among the few studies exploring reasons for long-term commitment, a recent survey conducted by MSF Sweden suggests that participation in peer-meetings and the opportunity to work in research or in their specialty area could contribute to higher retention among medical doctors [14]. An exploration of the experiences of retained humanitarian health workers can help clarify the aspects that facilitate humanitarian work overseas and thereby contribute to mitigating the high turnover rates in the sector. The aim of this study is therefore to identify reasons for continuous engagement in humanitarian missions by experienced humanitarian health workers.

### Theoretical framework

The analysis was inspired by van Gennep’s theory on the three-phased ‘Rites of Passage’ of life changes (1960) [15]. Life-cycle rituals mark the transition from one stage of life into a new one, such as birth, marriage and death [15]. They are often accompanied with ‘visible’ celebrations [15]. In modern societies rituals can also mark changes in more ordinary aspects of life, such as an academic or professional achievement [16–18]. They have been incorporated into the practices of institutions such as the United Nations (UN) and the Norwegian Government, to support individuals in making transitions and accomplishments meaningful [19–21]. The three-phased rite of passage structures the movement an individual undergoes from an old to a new role [15]. In the first phase – separation – the initiate experiences a physical, emotional or symbolic separation from the ‘old’ life. This is followed by a transitional phase where the individual is ‘in-between’ two roles which entail the acquisition of new skills [15,22]. During the third phase – incorporation- the individual re-emerges in the community in his/her new role [15]. The theory of rites of passage has been utilised in previous studies to understand the experiences of individuals undergoing life course transitions [16,23–26]. In this study, the rite of passage model was applied to the interview data to help capture and describe the changes humanitarian health workers experienced during each mission and over time.

Elements of the self-determination theory also lend to the framework of this study [27]. More specifically, the proposition that individuals have an inherent need to experience feelings of autonomy, relatedness and competence. These reflect, respectively, the need to experience a sense of choice and meaningfulness pertaining to own actions, to feel connected to others across contexts, and an urge to achieve efficacy through development of new competencies [27].

## Methods

### Study design and selection of participants

Semi-structured interviews were conducted with humanitarian health workers from the Swedish section of Médecins Sans Frontières. Within the section, reenlisting for new missions was reported to be more frequent among nurses as compared to other groups of health workers [14]. The study focuses specifically on this group of professionals as typical examples of healthcare workers, who seek to combine clinical work in their home countries with short- or long-term medical humanitarian missions. Retention was defined in terms of workers who had been taking on assignments in the field, and that were still registered as active in the pool of workers available for deployment. Nurses who had been to the field at least three times with MSF Sweden were purposively sampled, to secure the inclusion of participants who could describe their motivations and personal experiences over time. To minimise potential recall bias, only nurses whose last mission had taken place within the last three years were selected. An additional inclusion criterion was that the nurses were located in the two major cities of Sweden at the time of data collection, thus allowing for all interviews to be conducted face-to-face.

The Human Resources Department of MSF Sweden initially emailed a study introduction to the 17 eligible nurses. Eleven nurses agreed to participate and six nurses declined due to time constraints during the study period. During the process of data collection, one of the nurses was employed in a management position at the Swedish office. She was excluded due to potential conflict of interests in the dissemination of study results. Participants were all women and nine had a post-graduate education in areas such as intensive care, anaesthetics, paediatrics and public health. In the participants’ profile (see Table 1), countries of deployment are not linked to individual participants to secure the anonymity of the nurses.

**Table 1.** Profile of 10 female nurses interviewed in the study.

Study participant		Length of interview (minutes)	Age group (years)	Number of missions	Length of first mission (months)	Countries of deployment (per study sample)
N1	75		30–39	3	9	Afghanistan, Bangladesh, Burundi, Chad, Democratic Republic of Congo, Ethiopia, Guinea, Haiti, Ivory Coast, Kenya, Liberia, Libya, Myanmar, Niger, Nigeria, Pakistan, Papua New Guinea, Philippines, Sierra Leone, South Sudan, Syria, Zambia, Zimbabwe
N2	87		30–39	7	9	
N3	103		30–39	8	3	
N4	78		30–39	8	6	
N5	100		40–49	3	6	
N6	110		40–49	3	3	
N7	95		40–49	5	5	
N8	79		40–49	3	7	
N9	81		40–49	7	6	
N10	76		50–59	3	2	

### Data collection

A semi-structured interview guide was designed to explore participants’ expectations of missions, their experiences of working and living in the field and the support they received over time. To help nurses to more easily recall the actual enlistment for the first mission, the guide comprised a series of introductory questions covering the initial perceptions of humanitarian work and trigger points inspiring them to participate in humanitarian missions. The first mission was discussed in some detail to enable nurses to recall and subsequently compare between missions. Some of the questions covering the experiences in the field drew inspiration from the self-determination theory and the need to experience feelings of autonomy, relatedness and competence across contexts [27]. Two pilot interviews were conducted after which minor revisions were made. All 10 participants were interviewed at a place of their preference such as a library, a hotel, a café or at the MSF Sweden office. The interviews were conducted by SA in English, audio-recorded and transcribed verbatim during the spring of 2015. Interviews lasted between 75 and 110 minutes. Open-ended questions were mainly used and relevant concepts that emerged during interviews, such as nurses’ experiences of self-transformation or challenges of reintegration after missions, were further explored in subsequent interviews. Data collection was carried out until there was sufficient depth of information and redundancy of data to meet the aim of the study.

### Ethical considerations and confidentiality

Ethical approval was granted by the MSF Ethics Review Board (1 March 2015). Informed written consent was given by all participants prior to interview start. Human resources staff only provided the initial contact to the participants. They were not informed of who had agreed to participate and had no access to the interview transcripts.

### Data analysis

Data analysis was an evolving process that started after completion of the first interview and continued throughout the data collection.

The 220 pages of transcribed data were analysed according to the methodology of content analysis as described by Graneheim and Lundman [28], with the support of NVivo 10 software. The analysis was data driven and codes developed inductively, by means of repeated reading of the transcripts and constant comparison with the units of data previously coded. Codes would reflect different aspects of the initial perception of humanitarian work, motivations and experiences in the different missions, as well as in-between missions. Codes were compared to find patterns between interviews, and the tentative sub-categories were discussed by two researchers and subsequently revised. At this stage, the concept of the self-determination theory – ‘need for autonomy, competence and relatedness’– was introduced to refine some of the sub-categories related to the experiences during missions [27]. However, this theory did not capture an important feature that emerged in the data, namely the social dimensions of transformation. Further theories were therefore explored in order to interpret and make sense of the data at a more abstract level. As with the self-determination theory, other motivational theories concerned psychological features to a larger extent, thus failing to capture the important shift identified in nurses’ accounts and the role of the social context. Because of its focus on the social context, and the parallels between the three-phases of the model and the spectrum of nurses’ experiences in humanitarian work, the rite of passage model was regarded as a good guiding framework for the subsequent interpretation and development of categories and the main theme [15]. This iterative process involved constant dialogue and co-operation within the research team, with meticulous revision of codes and rereading of transcripts to secure that relevant data had not been inadvertently excluded.

## Results

In reflecting upon the changes that nurses had experienced during their participation in humanitarian work, we observed resemblances with the three phases of the rite of passage model. Findings are presented under the main theme ‘Constructing and sustaining new roles’. The theme is built on three categories: separation, transitional period and post-experience reincorporation, which are analogous to the three phases of a rite of passage. Each category illustrates a distinct stage of participants’ involvement in humanitarian work: from the individual motivations for deployment, to participants’ experiences in the different missions, to the post-assignment period. The relationships between the theme, categories and sub-categories are presented in Table 2.

**Table 2.** Theme, categories and sub-categories in the content analysis of nurses’ perspectives on remaining humanitarian health workers.

Theme	Category	Sub-category	
Constructing and sustaining new roles	Separation		Self-focused values as motivators
			Altruistic values as motivators
	Transitional period		Vulnerability
			Role-shifting
			Support of competence and autonomy
	Post-experience reincorporation		Struggling in-between two worlds
			Limited social mechanisms for maintaining change
			Individual coping strategies for maintaining change

### Separation

It was such a fantastic feeling that… little me, from a small town, never been to Africa before, that I was actually going to be in a refugee camp. (N9)

When asked about their motivations to participate in the different missions, all participants identified multiple reasons, some rooted in their personal preferences and needs and others related to the desire to help others. Altruism was expressed in terms of an initial and continuous sense of personal responsibility, compassion and urge for justice and equality. In this respect, a nurse who had been on eight missions commented:

My parents gave us…what I try to give to my children… That every person has the same value, we are all the same. And maybe that’s where it started. And that is what going away for me still is. To try to make the world a little bit more equal, so that children are not dying of malnutrition, because it’s not worthy to die of the malnutrition in 2015 when we have food! (N3)

Contrary to the consistency of altruistic factors, the personal motivations of the nurses changed over time. For initial participation in humanitarian work, they were interested in discovering new things and looking to experience adventure and feelings of worthiness, whereas for subsequent reenlistments nurses were looking to continue to challenge themselves and to enjoy the variety of emotions and stimuli from fieldwork. Also, participants cited the opportunities for career development as important to their continuation in humanitarian missions.

### Transitional period

Three dimensions captured the experiences of change during field missions: Initial period of vulnerability, role-shifting and support of competence and autonomy.

Feelings of fear and uncertainty prevailed when participants described their first humanitarian mission. Participants did not believe themselves to have sufficient knowledge and skills to fully live up to the mission requirements and many were worried about the challenges of communication and cooperation. In the field, three main factors increased feelings of vulnerability: limited guidance, lack of meaningfulness and unmet expectations. The majority were disappointed that the work was less ‘hands on’ than expected or that they were more disconnected from the patients than they had anticipated.

The sub-category ‘Role-shifting’ illustrates the subsequent changes that nurses experienced, both as individuals and professionals, during their stay in disaster-affected areas. Role-shifting contained several aspects: changes in personal character, changes in work-related skills and feelings of relatedness. One nurse who had been on seven missions explained what she felt after her second mission:

But then, this self-development, that came later on…and I think it is a fantastic part of this kind of life! I think…maybe I worked another mission, and then … you know, you start to believe much more in yourself, you can look back on things that you’ve been through and you can rely on that. (N9)

Many participants emphasised how they felt their personal values, beliefs and consumption habits became significantly different during the missions. Other types of changes in personal character related to recognising personal limits and adapting to very different working conditions through new coping strategies (see Table 3).

**Table 3.** Quotes illustrating changes in personal character of sub-category ‘Role-shifting’.

Code	Main sub-codes	Typical quotes
Changes in personal character	Positive thinking	That you’re actually able to make sure that every small good thing is a massive good thing. Just to concentrate on the good things, rather than the bad things that you see happening. I think I learned that. (N2)
	Recognise the limits	I felt this a lot, after my first two missions, like a comfortable feeling within myself that ‘Well, this is me, I can’t do much more. These are my limits'.(N7)
	Flexibility	I learned that I can be very flexible. And that is a big advantage in MSF. That you adapt, to very changing situations, with very changing tasks. (N5)
	Calmness	You are so challenged, both on a personal and professional level, when you’re out in the field…so it prepares you! I have become quite calm, I would say. It’s like…there are not a lot of things that stress me. (N7)

Role-shifting also covered changes in work-related skills, such as an enhanced ability to cooperate, to master different tasks and take on managerial positions. Feelings of relatedness were also developed, with many nurses emphasising a sense of sharing similar values and beliefs, both with the other staff and the local population. Nurses experienced strong bonding to and friendships with the international team. Overall, sharing deployment experiences with their peers, particularly those starting out in humanitarian work, conferred a sense of not feeling alone and was perceived as fundamental to the success of their deployment.

Two main sources of support were seen as decisive to the self-transformation process experienced in the field: conditions supportive of competence and of autonomy. Participants were generally pleased with the training, the encouragement of their self-development and the constructive feedback on their performance. Associated with nurses’ sense of autonomy were feelings of freedom, choice and meaningfulness when working in the field. For instance, participants appreciated being able to perform their tasks without having too strict supervision or having the opportunity to be involved in decisions, even during their first missions. Many highlighted that working close to the local population conferred increased meaningfulness to their assignment:

It’s easier if you, on a daily basis, see the children or see the mothers…see with your own eyes that you are actually saving people there. And then, compared to when you work as a coordinator, it’s more statistics, you see it on paper what we do …but not really in reality. (N7)

The experience of self-transformation that accompanied the transitional period culminated with participants having a new way of thinking of themselves as better skilled and more confident, with an increased capacity to relate to others and having redefined personal values.

### Post-experience reincorporation

#### Struggling in-between two worlds

Coming home from missions was described using words such as ‘clash’, ‘hard’ and ‘long’. The following quote illustrates what a nurse felt after her first deployment:

In the field you are really living it and breathing it! It took really long time, when I was at home, to actually think about anything else except (country of mission). At work, every break, I went down to the library, reading about the news in (country of mission). It takes a while to become open again to normal life in Sweden. (N5)

A common topic discussed by nurses was the sense of feeling unfulfilled at their workplace in Sweden. Contrary to what they experienced in the field, the variety of emotions and the meaningfulness of the work were often missing. Also, after experiencing the field it became difficult to understand the consumption habits, needs and daily worries of people in Sweden.

#### Limited social mechanisms for maintaining change

Four distinct sources of social support – MSF, the Swedish employer, family/friends at home and peer support – influenced how nurses adapted back home. Participants discussed how these actors could help them, but also gave examples of when these support structures were not fully in place. In general, nurses were positive about the support from the MSF Swedish office, where they felt they were listened to and taken care of, particularly during the individual debrief post assignment and the ‘Returnee Workshop’, held twice a year for all returned field workers. In spite of nurses’ requests to get absence of leave from current Swedish employers, the majority were disappointed with the inflexibility of their employers and found it difficult to combine fieldwork with a permanent position at home. Support from family and friends were among the most discussed features. Overall, participants mentioned a lack of understanding of their life choices and highlighted the difficulties of sharing field experiences with those closest to them, either due to a perceived lack of interest, or because others did not know how to inquire about and interpret such experiences. One nurse explained how she felt after returning home from her first mission:

I had a very tough time coming home, that’s for sure. It was very tough. I didn’t know whom to talk to (…) I felt that people, friends or family… they were curious, to start with, but then (pause) I think I wanted them to ask more questions, or I wanted to talk about it much more! I think people lose their interest or they don’t know what to ask about. (N9)

Instead, participants chose to discuss their experiences with MSF peers, with whom they felt understood.

#### Individual coping strategies for maintaining change

Back home, nurses strived to strengthen their skills for subsequent deployments, either with postgraduate education or working in particular medical areas that they could associate with field work. Some chose not to have a permanent contract at home, in order to be able to accept new missions without restriction. A significant number mentioned that they avoided sharing the difficulties experienced in the field with family and friends, as this might have negative implications for future reenlistments. Also, some participants spoke about the importance of accepting the consequences of their own choices, such as the decision to pursue a career in humanitarian aid. For example, one nurse commented:

Generally in Sweden, or maybe in the Western world, people don’t want to choose. They want the typical career, they want a family, a well-trained body… they want everything! They don’t want to say ‘Ok, if I do this, I cannot do that’. And I think that you do have to choose. If you do something, maybe you have to choose not to do something else! (N5)

The interviewed nurses had thus all identified ways of choosing humanitarian work as one part of their lives which were meaningful to them.

## Discussion

To our knowledge, no previous study has applied the rite of passage model to understand the social dimensions of transformation among humanitarian health workers and the significance of these to long-term commitment in the sector. The model illustrated the initial fears and uncertainties of the nurses, the subsequent change in their roles and how they became more confident, flexible, able to relate to others and sensitive to cultural context. It was especially helpful in understanding the subsequent problems of reintegration and maintenance of new roles and skills gained from the missions.

Separation is necessary to achieve change in the van Gennep’s sense of the term and findings from the study highlight factors that preceded and prompted nurses’ involvement in missions, and thus their experiences of transformation and role change during missions. Consistent with previous findings on reasons for initiating humanitarian work, nurses were initially attracted by the idea of assisting others in need and to experience novelty and feelings of worthiness [5,6]. A previous study with experienced humanitarian workers cited altruistic values as motives for continuous engagement [8]. An important finding of the present study is that continuation in the sector is also tied to reasons of personal development and satisfaction.

The ‘transitional period’ was initially characterised by nurses’ feelings of uncertainty, vulnerability and low self-esteem. Similar findings have been reported in studies on other types of life course transitions such as men’s passage to fatherhood, which was associated with sentiments of vulnerability and uselessness [26] and student nurse practitioners’ acknowledgement of feeling invisible and insecure about their capacity during the ‘journey’ of their clinical degrees [16]. Consistent with trends observed in the two previously mentioned studies, the initial period of vulnerability was followed by a gradual ‘transition’ to their new roles [16,26]. Feelings of competence, autonomy and relatedness that nurses developed in the field were key to the emergence of the new role as humanitarian health worker. Reported support of competence – such as proper training and constructive feedback – and of autonomy – in terms of involvement in decisions and the meaningfulness of the assignment – are consistent with key techniques referred to in the literature as encouraging feelings of competence [29] and autonomy [29,30], respectively. The experienced feelings of relatedness, both with other staff and the local community members, were attributed particular relevance in coping with the vulnerability of the ‘transitional period’. In previous research conducted in healthcare [16] and military [31] settings, it was reported that this socialisation process and group bonding can ease this phase and contribute to successful transitions. The role of social relationships in international aid work was also discussed in a previous paper on aid work [9]. The author outlines that personal relationships are instrumental to improving the well-being of the workers and the delivery of aid [9]. In the same line, findings from previous studies on organisational socialisation emphasise the importance of relationship-building to employees’ adjustment and commitment to a new organisation [32,33].

Reintegration in the home-community posed some constraints to the previous successful role transition. The same limitation was highlighted in a study on outdoor education programmes and their effectiveness as rites of passage [22]. The author reported the lack of an adequate post-trip structure to be one of the main drawbacks of such programs, leading to a failure in supporting successful transitions [22]. The idea that nurses should not undertake their rite of passage alone and that the community is central in reinforcing workers’ new role and achievements also has support in previous literature on career theory [17]. The authors state: ‘no psychological role transformation will be successfully maintained if it is not reinforced by others around us’ [17, p.410]. Findings from a study with health professionals who were about to initiate in humanitarian work reinforce this argument: participants expressed concern with the possible lack of recognition from community members when returning home [5]. In the face of a fragile system of reincorporation, the overall dissatisfaction about their home-community context, the support from their peers and the individual coping mechanisms these nurses developed back home may to an extent explain why study participants continued to have the eagerness to join new missions. However, this may not be the case with many of the individuals that, despite experiencing successful transitions during deployment, return to a community not able to fully support their changes. As with the outdoor education programmes [22], it is likely that workers return to their former roles in the community, rather than continuing their humanitarian endeavours.

The findings on nurses’ problems of reintegration in the home country indicate that humanitarian organisations should focus on having an appropriate system of support for returned workers, by encouraging feelings of relatedness through peer networking and a more active participation of relatives and friends in some of the organisations’ practices. Such an approach may open the door to an increased understanding on the role and choices of these workers. In the UN and the Norwegian Government, greater attention has already been given to rites, ceremonies and family involvement [19–21]. For the UN, such events present opportunities for their workers ‘to reflect on what it means to serve the United Nations’ [19, p.42], whereas the Norwegian Government introduces its ‘In Service for Norway’ as a means ‘to reinforce society’s recognition and care for personnel who serve in international operations [21, p.6]. These two cases exemplify how organisations can celebrate achievements of their employees, and enhance emotional bonds, both within the organisation and within the community. Also, the nurses’ desire to add on their expertise and to engage in personally meaningful work back home, suggests that home-based employers could also facilitate the reintegration of returned workers. Supporting further training, education and the participation in work where nurses could exercise some of the skills acquired during missions, could possibly contribute to promoting their feelings of autonomy and competence.

### Methodological considerations and further research

The study is based on interviews with 10 participants that all expressed a high degree of interest in the topic and willingness to reflect on their experiences. It was observed that no new themes or codes were obtained in the two final interviews which indicated saturation on the main reasons for retention.

It is of note that a study sample of female nurses poses limitations in terms of transferability to other professions of humanitarian workers and limits possible explorations of the role of gender. We encourage further research on humanitarian worker retention that adds diversity with respect to different organisations, professional backgrounds and gender. Also, this study only reflects on the perspectives of international staff, who are subject to distinct challenges when compared to national staff. Research on the experiences and needs of humanitarian workers could be extended to national staff, as they constitute the majority of the workforce of humanitarian organisations.

This study did not include those who discontinued their engagement in humanitarian health missions. This was a deliberate choice to secure a study sample of cases that could best contribute to the aim of the study. Yet, assessing in-depth the experiences that deterred continued involvement in missions among those who have left the sector could provide further inputs into the formulation of retention strategies.

Finally, it is worth highlighting that this study has generated important insights into the specific challenges of reintegration after missions, suggesting the relevance of pursuing this avenue further in future studies on retention in the sector.

## Conclusion

The motivations of humanitarian health workers that have been identified indicate that both the commencement of humanitarian health work and the willingness to go on further missions are encouraged by a combination of personal goals and the principal of assisting others. The findings show that what individuals experience and the assistance they receive during deployment and after missions have great significance to long-term commitment. In particular, the challenges nurses experienced with reintegration highlight the need for a home community able to fully acknowledge and support their new achievements and responsibilities. These findings can assist humanitarian organisations to project how they can develop or strengthen strategies to facilitate long-term commitment in the sector, which can ultimately improve the delivery of humanitarian assistance.

## Paper context

Despite the current need for greater retention of humanitarian workers, there is little evidence to inform organisational practices designed to facilitate long-term commitment in the sector. Drawing on interviews with experienced humanitarian health workers, this study provides insights into their motivations, needs and individual experiences over time. Findings emphasise the major role of supportive social environments, during and after field missions. The study can contribute to the development of actions that lead to improved staff retention.
